# RNA modification regulator DDC in endometrial cancer affects the tumor microenvironment and patient prognosis

**DOI:** 10.1038/s41598-023-44269-2

**Published:** 2023-10-23

**Authors:** Huai Zhao, Chuang Shi, Guoguang Zhao, Jiamin Liu, Xi Wang, Jie Liang, Fangmei Li

**Affiliations:** 1 Shengjing Hospital of China Medical University, 110001 Shenyang, Liaoning China; 2 Guangming Community Health Service Center, 101127 Shunyi District, Beijing, China; 3 Zigong First People’s Hospital, 643099 Zigong, Sichuan China; 4 The First Hospital of China Medical University, 110001 Shenyang, Liaoning China

**Keywords:** Bioinformatics, Cancer

## Abstract

Uterine corpus endometrial carcinoma (UCEC) is infiltrated by immune cells, which are involved in the growth and proliferation of malignant tumors and resistance to immunotherapy. This study suggested that RNA modification regulators played an important role in the development and prognosis of UCEC. Many studies confirmed that RNA modification played an essential role in tumor immune regulation, and abnormal RNA modification contributed to tumorigenesis and cancer progression. Based on the RNA modification regulatory factors, the UCEC samples from TCGA (The Cancer Genome Atlas) were classified into two clusters, namely Cluster A and Cluster B, using unsupervised consensus clustering. We obtained DEG (differentially expressed genes) between the two clusters, and constructed a risk model of RNA modification-related genes using DEGs. Cluster A had lower RNA modification regulatory factors, richer immune cell infiltration, and better prognosis. The differentially expressed genes between the two clusters were obtained, and these genes were used for modeling. This model divided patients with UCEC into two groups. The low-risk group had better immune infiltration, and the ROC (receiver operating characteristic) curve showed that this model had good predictive efficacy. The low-risk group had a better response to immunotherapy by immune checkpoint prediction. We obtained the key gene l-dopa decarboxylase (DDC) through the intersection of LASSO model genes and GEO dataset GSE17025. We evaluated the potential biological functions of DDC. The differences in the expression of DDC were verified by immunohistochemistry. We evaluated the relationship between DDC and immune cell infiltration and verified this difference using immunofluorescence. Cluster A with low expression of RNA modification regulators has better prognosis and richer immune cell infiltration, therefore, we believed that RNA modification regulators in UCEC were closely related to the tumor microenvironment. Also, the risk score could well predict the prognosis of patients and guide immunotherapy, which might benefit patients with UCEC.

## Introduction

The tumor microenvironment includes, including immune cells, stromal cells, including extracellular matrix (ECM), blood vessels, lymphatic vessels, cytokines and other non-cellular components^1,2^. The most important cells are infiltrating immune cells^3^. Moreover, stromal cells exert a considerable influence on tumor metabolism, evasion of the immune system, and resistance to treatment^1,4^. Immunosuppression is an important feature of tumor microenvironment (TME), which contributes to the occurrence and development of tumors. Immunosuppression is mainly to recruit or generate a variety of inhibitory immune cells, such as CD4+ regulatory T cells (Tregs) and dendritic cells (DCs), so as to evade immune surveillance^5^. Immune cell infiltration is closely related to tumor growth and invasion, and is influenced by many factors. RNA modifications have been identified to exhibit close connections with the immune response. These alterations play a pivotal role in the promotion of tumor growth by controlling the manifestation of immune checkpoints and impacting the secretion of immune-related molecules. They empower malignant cells to circumvent the immune system^6,7^. In this study, both the unsupervised clustering analysis of RNA regulators and the risk score of RNA modification-related genes indicated that RNA modifications promoted immunosuppression within the EEC tumor microenvironment (TME).

Uterine corpus endometrial carcinoma (UCEC) is a common gynecological malignancy^8^. Obesity, age, family history of cancer, and increased estrogen levels are all important risk factors for endometrial cancer^8,9^. The primary treatment modality for endometrial cancer encompasses the comprehensive surgical procedures of total hysterectomy and bilateral salpingo-oophorectomy^10^. Targeted chemotherapy strategies represent a novel direction in the treatment of endometrial cancer^8^. However, the prognosis of advanced patients is poor^11^, which still needs better therapy development.

Research findings indicate that there are notable changes in the expression levels of different factors responsible for N6-methyladenosine (m6A) regulation in EC. It is crucial to highlight that the development and progression of EC heavily depend on abnormal m6A methylation and the presence of associated regulatory factors^12^. This study revealed that RNA modifications have an adverse impact on the prognosis of uterine corpus endometrial carcinoma (UCEC) and profoundly influence the immune infiltration in UCEC.

Dopa decarboxylase (DDC) is a crucial enzyme involved in the biosynthesis pathway of catecholamines, which relies on the presence of pyridoxal phosphate (PLP). This enzyme is primarily responsible for the synthesis of important neurotransmitters like dopamine (DA) and serotonin (5-HT)^13^. Biogenic amines contribute to various biological processes, including angiogenesis, cell proliferation, differentiation, and apoptosis^14–16^, implying the potential significance of DDC in the pathobiology and progression of cancer. DDC serves as a diagnostic marker for neuroblastoma^17^. Reliable evidence has linked DDC to the prediction of peritoneal metastasis in patients with gastric cancer^18^. Notably, the expression of DDC is significantly elevated in prostate cancer tissues compared to benign prostatic hyperplasia. This upregulation is strongly associated with prostate invasiveness, as well as the pathological stage and Gleason score of patients^19^. These investigations emphasize the potential importance of DDC in the oncobiology of cancer and its ability to forecast patient prognosis. We intersected the differentially expressed genes (DEGs) in endometrial carcinoma and adjacent tissues with the LASSO model genes to obtain the DDC gene and verified the differential expression of DDC in our patient cohort through immunohistochemistry (IHC). We found that the low expression of DDC was associated better prognosis. DDC is a risk factor for UCEC and a key factor in the risk model of RNA modification-related genes.

## Material and methods

### Data of UCEC

Sequencing, clinicopathological information, and related data of 537 patients with endometrial cancer and 34 normal patients were extracted from the TCGA database (Appendix 1: Sequencing data and clinical information of UCEC from TCGA source). The dataset GSE17025 (79 UCECs and 12 normal endometrial tissue) from the GEO database was applied to seek the signature DEGs (Annex 2: Sequencing data from the GEO dataset GSE17025). A total of 125 RNA modification regulators were identified from the published literature^20,21^ (TCGA data through https://xenabrowser.net/datapages/. GEO data obtained through https://www.ncbi.nlm.nih.gov/geo/query/acc.cgi?acc=GSE17025).

### Unsupervised clustering based on RNA modification regulator

In order to observe the differential expression patterns of RNA modification regulatory factors in endometrial cancer samples. The ConsensusClusterPlus package (ConsensusClusterPlus 1.64.0) was used to extract 80% samples and 100% genes simultaneously, repeated 1000 times to ensure classification accuracy. Unsupervised consistent clustering was performed on 537 UCEC patient samples based on 125 RNA modification regulators. Based on the clustering results, the optimal consensus matrix K = 2 was selected and divided into categories A and B. A pheatmap package was used to cluster RNA modification regulators in both groups to construct a visual heatmap. The DEG analysis of the two types was performed using the package DESeq2 (DESeq2 1.40.2) to compare pathway differences between the two subtypes. DEGs were obtained based on the count data of the two groups, with log2FC > 1 and adj.p.value < 0.05. The clusterProfiler package (clusterProfiler 4.8.1) is used to perform gene ontology (GO), Kyoto Encyclopedia of Genes and Genomes (KEGG), and Gene Set Enrichment Analysis (GSEA) enrichment analysis for the differentially expressed genes (DEGs) of these two subtypes^22–24^. The survival package (Survival 3.5.5) was used to calculate the survival of each subtype as a function of clinical prognosis based on the expression profile data of UCEC tumor samples downloaded from the TCGA database and clinical data to explore the connection between the two subtypes and clinical prognosis. The clustering results were used as the basis for grouping, and the Kaplan–Meier plot was drawn. A *P* value ≤ 0.05 indicates a significant difference.

### Evaluation of the immune landscape between two subtypes

The signature genes were downloaded from the Supplementary Table 1 of the CIBERSORT paper (https://www.nature.com/articles/nmeth.3337#MOESM207), and the following matrix was obtained and saved as TAB segmented txt (" LM22.txt "). Since the sum of all cell fractions in a single sample in this algorithm was 1, the ratio of each immune cell to all immune cells could be obtained. Therefore, CIBERSORT (R package e1071 1.7.13, parallel 4.3.1, preprocessCore 1.62.1) was applied to evaluate the relative infiltration of 22 types of immune cells in the 2 cluster subtypes. The ssGSEA algorithm calculated the enrichment fraction according to the absolute rank conversion of the gene expression amount in the sample, which was repeated 1000 times to get the final enrichment fraction. Therefore, ssGSEA (R-packet GSVA 1.48.3) was applied to calculate the absolute infiltration of immune cells in the two groups of RNA-modified subtypes. The " estimate 1.0.13" package was used to assess the immune microenvironment of all tumor samples, including stromal score, immune score, estimated score, and tumor purity score. The estimated score was a combination of stromal and immune cell scores. The intergroup differences between the two RNA-modified-subtype patient cohorts were then compared.

### Construction of the prognostic model

The samples were grouped according to the two RNA-modified subtypes, and then the DEGs were screened out. Subsequently, Cox univariate regression analysis was performed, and the prognostic genes were obtained with *P* < 0.001 as the cutoff value. A total of 537 UCEC samples (TCGA) were randomly assigned to the training group (*n* = 430) and the test group (*n* = 107) at a ratio of 4:1. We used the glmnet (glmnet 4.1.8) package to build a LASSO regression model based on the obtained prognostic genes and the expression profile data of the training group (tenold cross-validation, 1000 iterations, random number seed set to 3) to obtain the connection between prognostic genes and prognosis. Further, *λ* = lambda.min (minimum cross-validation error *λ*) was used to get the model coefficients. The risk score of the training group was calculated according to the obtained model coefficient and corresponding gene expression profile. The median of the sample risk score was taken as the cutoff value, and the training group was divided into high- and low-risk groups. The "ROCR 1.0.11" package was used to judge the predictive ability of the model, and the ROC curves and corresponding area under the curve (AUC) values for 1, 3, and 5 years were obtained. Then, the obtained model was added to the test group data, and the ROC curve and AUC value were used to judge the generalized ability of the model. Finally, the model was applied to all 537 UCEC datasets, the AUC values were obtained, and the Kaplan–Meier graph was drawn using the R package “Survival 3.5.5”. Univariate and multivariate Cox regression analyses were performed based on the LASSO risk score, sample stage, grade, and age to assess whether the risk score was an independent prognostic factor.

### Immune microenvironment analysis between high- and low-risk groups

CIBERSORT, ssGSEA, and Xcell were used to compare the proportion and absolute value of immune cell infiltration between high- and low-risk groups. The "estimate 1.0.13" package was used to evaluate stromal cell score, immune cell score, comprehensive score, and tumor purity score for the high- and low-risk groups. The ggplot2 package was used to visualize the scoring data. We plotted Kaplan–Meier graphs for various scores using the R package “Survival 3.5.5” to seek the connection between these scores and prognosis. We observed the expression of a series of immune checkpoints between normal and tumor samples and between high- and low-risk groups, again using ggplot2 (ggplot2 3.4.2) package visualization. The networkD3 (networkD3 0.4) package was applied to construct a mulberry map according to the clustering, LASSO model, staging, grading, and survival among samples, which was used to visualize the internal relationship among clustering, LASSO model, staging, grading, and survival.

### Establishment of a nomogram

A Cox regression model was constructed with grouping, staging, and grading the LASSO model to obtain a nomogram so as to improve the efficiency of the model.

### Identification of key genes

The DEGs were analyzed according to their expression profiles in the TCGA tumor and normal samples. The GEO database GSE17025 was used to screen out the DEGs using the "limma 3.56.2" package, and the intersection of the two groups of DEGs and LASSO model genes was finally obtained.

### Verification of key gene expression

Tumors and paired normal thyroid tissues were obtained from a cohort of 60 patients with UCEC. The tissues were fixed in formalin. The IHC staining was implemented strictly according to the instructions of the reagent manufacturer (MXB, China). The antibodies against DDC were obtained from Abclonal (1:50, Abclonal, China). The IHC results were scored independently by three experienced pathologists based on the depth and area of the staining.

### Analysis of the potential biological functions of key genes and the relationship between key genes and immune infiltration

The potential biological functions of key genes were analyzed by GO and KEGG enrichment analyses. The TCGA patient cohort was divided into two groups, with the median DDC expression as the boundary. CIBERSORT and ssGSEA were used to compare the proportion and absolute value of immune cell infiltration between the two groups.

### Verification of the influence of key genes on immune cell infiltration

The UCEC data downloaded from the TCGA database were processed with “e1071 1.7.13”, “parallel 4.3.1”, and “preprocessCore 1.62.1” packages to evaluate the effect of DDC on immune cell infiltration. The patients were divided into two groups according to the median expression level of DDC, and the immune cell infiltration of the two groups was analyzed. The tissue sections were immersed in the antigen repair solution, and the antigen repair was performed in a microwave oven. The tissue was coated with 3% bovine serum albumin for 60 min at room temperature. The blocking fluid was removed, and regulatory T cells with the FOX3P antibody (1:100, Abclone) were added and incubated in a wet box at 4 °C for 16 h. The sections were rinsed with phosphate-buffered saline (PBS) and incubated with a fluorescent secondary antibody (Abbkine, American) for 30 min. Then, they were rinsed three times with PBS. They were stained with DAPI (4′,6-diamidino-2-phenylindole) (Solarbio, China) for 4 min. The washing was repeated. Complete stained slides were sealed with a quench-resistant sealant (Solarbio, China). The images were taken with a fluorescence microscope (Nikon, Japan). The average Treg density for each sample was calculated.

### Statistical analysis

Statistical analysis was performed using R 4.2.0 and GraphPad Prism 8.

## Results

### Identification of RNA-modified isoforms in UCEC

**Figure 1:**
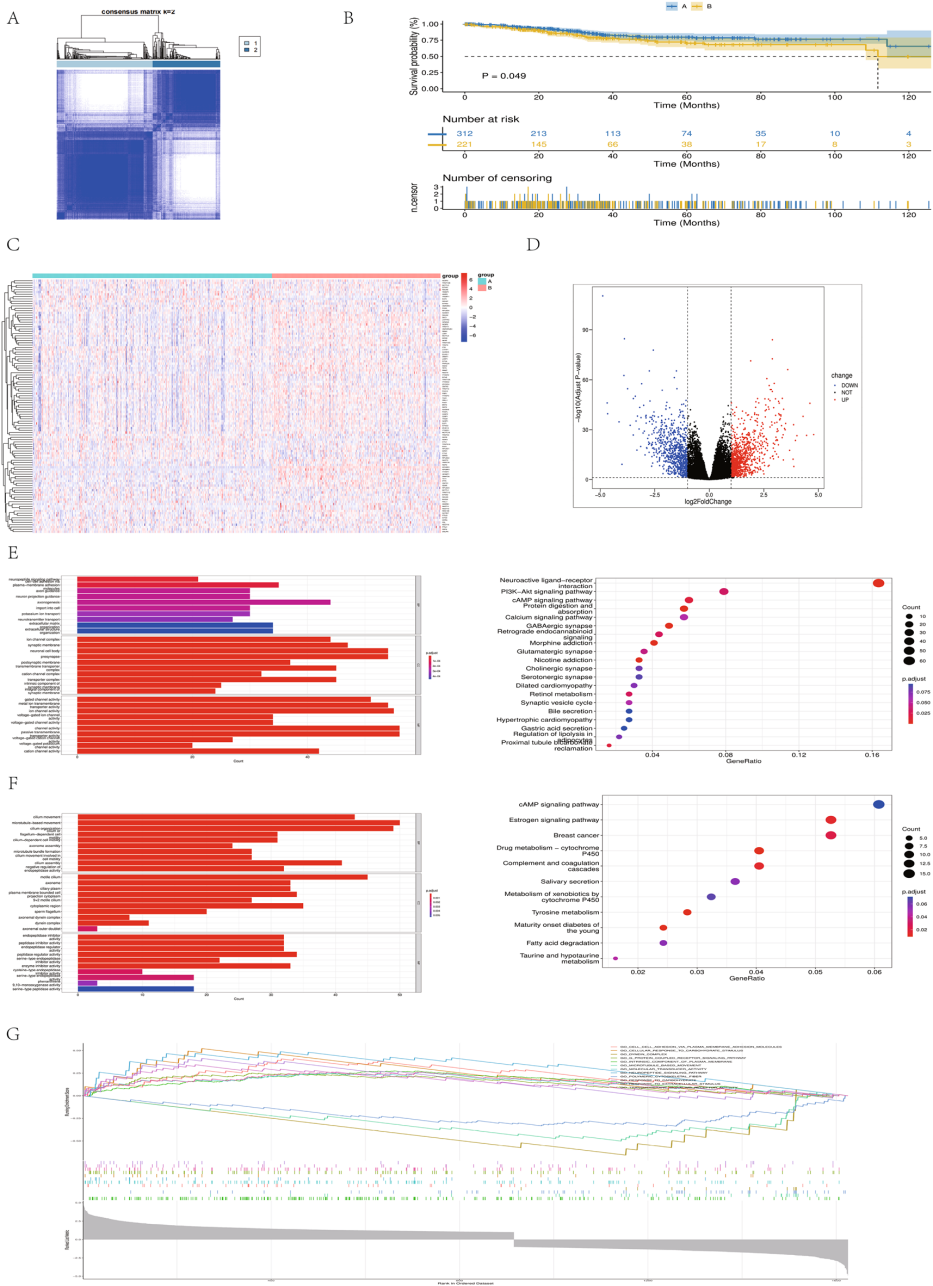
HYPERLINK "sps:id::fig1||locator::gr1||MediaObject::0"(**A**) Cluster group of K = 2 patients in the TCGA database. (**B**) Survival analysis Kaplan–Meier curve of RNA modification classification in the TCGA cohort. (**C**) Cluster heatmap of all RNA modification–related genes in the TCGA cohort. (**D**) Differential gene volcano map of RNA modification classification in the TCGA cohort. (**E**) GO and KEGG enrichment analysis of highly expressed genes in RNA-modified group A. (**F**) GO and KEGG enrichment analysis of highly expressed genes in RNA-modified group B. (**G**) GSEA analyzes the pathway between two RNA modification isoforms.

Two different RNA modification modes were identified by unsupervised clustering according to the expression of RNA modification regulators in TCGA: type A (315 cases) and type B (222 cases) (Fig. 1A). The blue line represents the prognosis of cluster A, while the yellow line represents the prognosis of cluster B. The survival analysis based on TCGA database UCEC clinical data showed that the survival advantage of group A was significantly higher than that of group B (Fig. 1B). Next, We generated a heatmap depicting the expression levels of RNA modification regulators in the two subtypes. (Fig. 1C). Most RNA modification regulators in group A tended to have low expression compared with those in group B. Later, with | LogFC |> 1 and padj < 0.05, 1721 DEGs were obtained in groups A and B (Annex 3; DEGs) (Fig. 1D). KEGG and GO enrichment analyses were conducted between the two groups. The results showed that PI3K-Akt, cAMP, calcium signaling pathway, ion channels, and other related other pathways and functions were enriched in group A. In group B, estrogen signaling pathway, fatty acid degradation, cytochrome P450-mediated metabolism, breast cancer, and other related pathways and micro-related functions were enriched. GSVA (Gene Set Variation Analysis) analyzed the differences in the pathways between the two clusters (Fig. 1E–G).

### Immune microenvironment of RNA-modified subtypes

**Figure 2:**
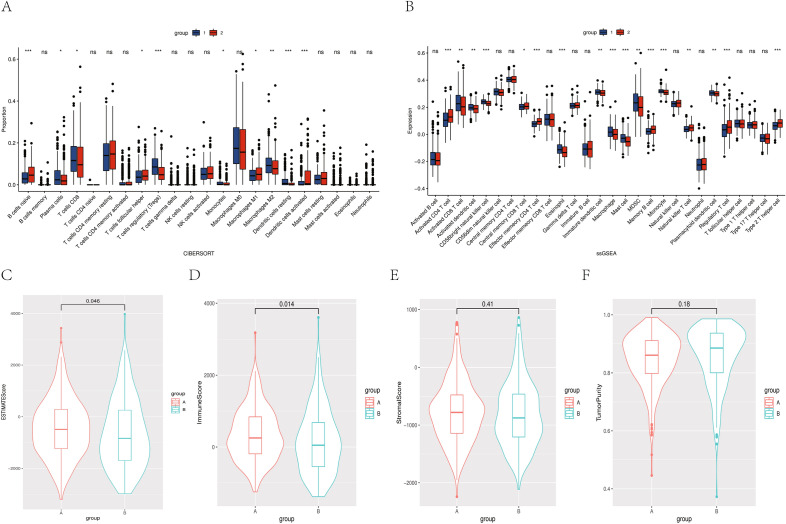
(**A**) CIBERSORT analyzed the immune cell infiltration landscape of the two groups of RNA-modified subtypes. (**B**) ssGSEA analyzed the immune cell infiltration landscape of the two groups of RNA-modified subtypes. (**C**–**F**) ESTIMATE score, immune score, StromalScore and TumorPurity of the two groups of RNA-modified subtypes.

CIBERSORT and ssGSEA were used to visualize the infiltration of different immune cells so as to investigate the difference in immune infiltration between the two RNA-modified subtypes. The immune infiltration of cluster A was characterized by plasma cells, CD8 T cells, Tregs, and M2 macrophages with high infiltration. The immune infiltration characteristics of cluster B were B cells, activated natural killer (NK) cells, M1 macrophages, CD4 T cells, and central memory CD8 T cells with high infiltration (Fig. 2A and B). We introduced the ESTIMATE algorithm, which could calculate the stromal score and immune score according to the corresponding stromal signature and immune signature in tumor tissues. The estimated score and tumor purity were obtained by the synthesis of the stromal score and immune score. Cluster A had a higher estimate score and immune score than cluster B (*t* test, P < 0.05), while the stromal score and tumor purity had no significant difference (Fig. 2C–F). This indicated that the immune microenvironments of cluster A and cluster B were significantly different. Cluster A contained more immune cell infiltration, which was perhaps related to the lower expression of RNA modification–related genes, and might also be the reason for the better prognosis of cluster A.

### Prediction of prognosis in endometrial cancer by RNA modification-associated risk models

**Figure 3:**
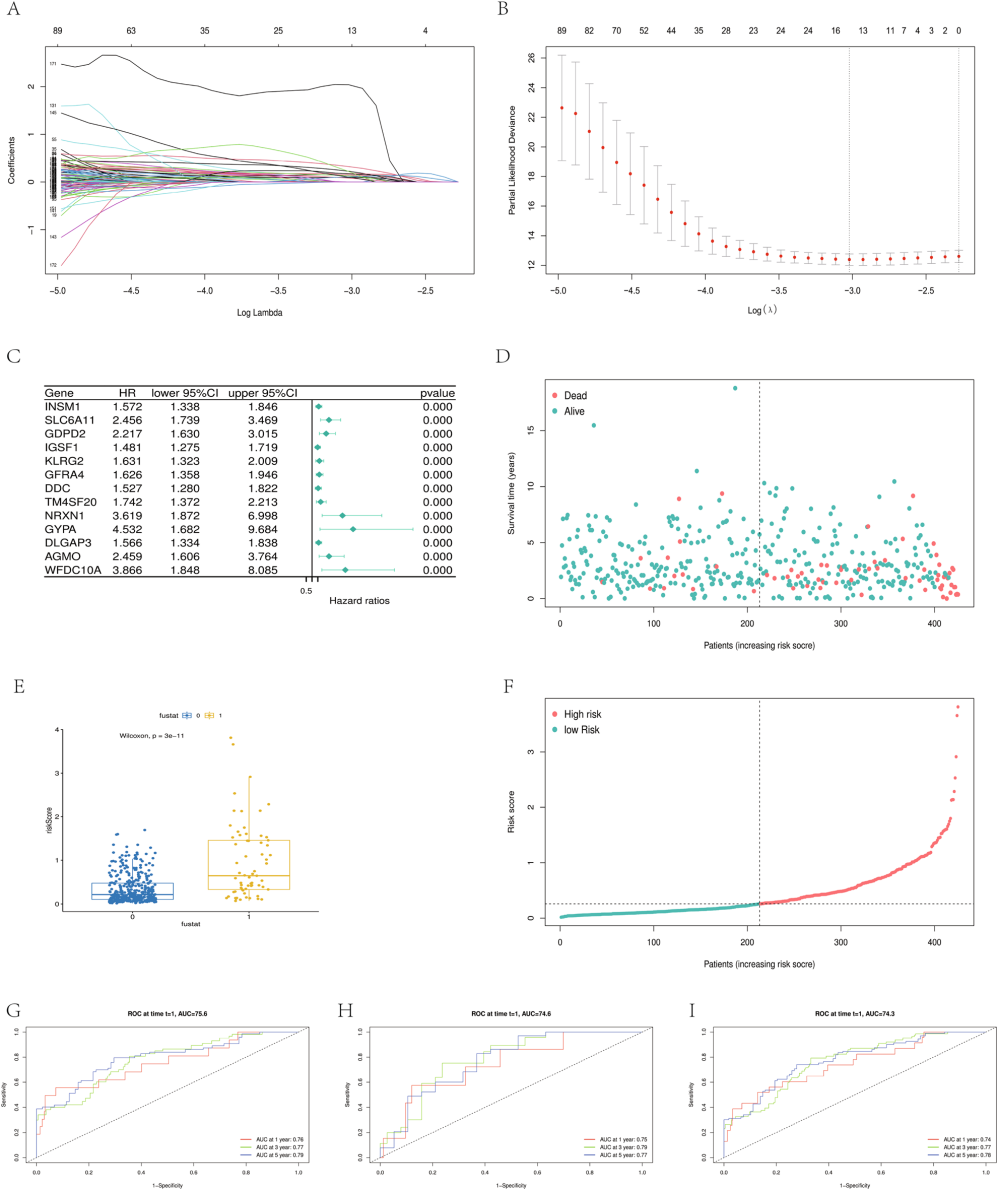
(**A**) LASSO regression model coefficient selection. (**B**) Thirteen RNA modification–related genes were selected using minimum λ criteria. (**C**) Correlation forest map between the expression of 13 prognostic genes and OS in the training group. (**D**) Risk distribution between the risk score and survival status in a training cohort. (**E**) Box graph of scoring differences among training groups based on the survival status. (**F**) Training group was divided according to the median risk score. (**G**) ROC curves of the training group after 1, 3, and 5 years (**H**) ROC curves of the test group after 1, 3, and 5 years. (**I**) ROC curves of the TCGA cohort after 1, 3, and 5 years.

DEGs between RNA-modified subtypes were obtained based on the expression profile data and overall survival (OS) information of endometrial cancer samples in the TCGA database, following which 187 key prognostic genes were screened by Cox univariate regression analysis with *P* < 0.001 as the cutoff value (attached information). All 537 patient samples containing OS information in TCGA were randomly grouped to obtain 430 patient samples in the training group and 107 in the test group. The LASSO Cox regression model was used to select the best prognostic genes from the key genes in the training group. After 187 key genes were included in the model, the RNA modification–related risk score model was built with 13 RNA modification–related signatures after using minimized lambda (Fig. 3A and B). Figure 3C shows the relationship between the expression of 13 final signatures and OS. The expression levels of all 13 RNA modification–related signatures were associated with poor prognosis. The RNA modification–related risk score was calculated as follows: RNA modification–related risk score = (0.095 × INSM1) + (0.321 × SLC6A11) + (0.148 × GDPD2) × (0.128 × IGSF1) + (0.080 × KLRG2) + (0.223 × GFRA4) + 0.176 × (DDC) + (0.128 × TM4 SF20) + (0.102 × NRXN1) + (2.039 × GYPA) + (0.106 × DLGAP3) + (0.170 × AGMO) + (0.219 × WFDC10A) (Annex 4; Risk score). Figure 3 clearly shows that the elevated risk scores were associated with poor outcomes. We divided the training group samples into two groups according to their prognostic outcome OS: 0 representing alive and 1 representing dead. As shown in Fig. 3E, the risk score in the dead group was significantly higher than in the alive group (*P* < 0.001). The training group was divided into high- and low-risk groups according to the median RNA modification–related risk score (Fig. 3F). The ROC curve for the training group was also drawn according to the prognostic model established between the two groups. This model was proved to have strong prediction efficiency, with the precision is 0.25, recall is 0.80, and the F1 score is 0.38 (Fig. 3G). We introduced the model into the test group and the full sample of TCGA to test its generality, and found that the AUC in the first, third, and fifth years in the test group was 0.75, 0.79, and 0.77, respectively (Fig. 3H). The AUC in the first, third, and fifth years for TCGA samples was 0.74, 0.77, and 0.78, respectively (Fig. 3I). This indicated that the model could be extended to other UCEC data and had good predictive power.

### Differences between high- and low-risk groups associated with RNA modification

**Figure 4:**
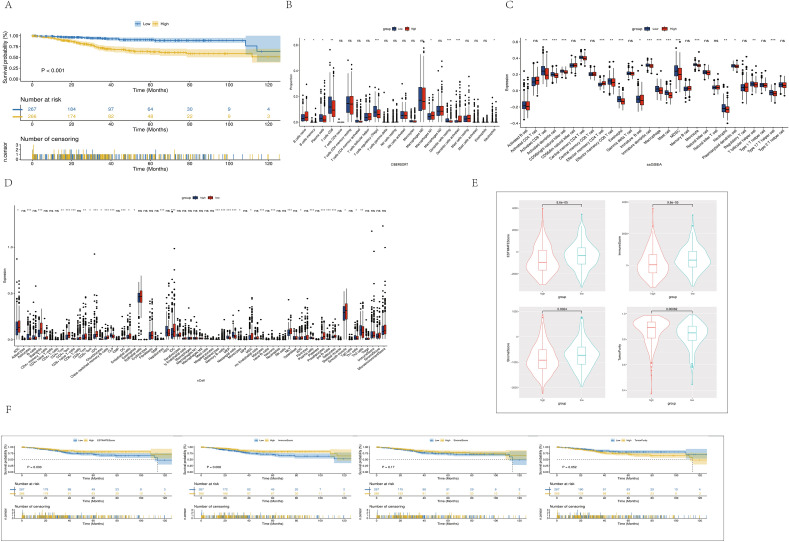
(**A**) Kaplan–Meier survival curve of OS between high- and low-risk groups in the TCGA. (**B**) CIBERSORT analyzed the relative proportion of immune cell infiltration. (**C**) Absolute value of immune cell infiltration in the TCGA by ssGSEA analysis. (**D**) XCell analysis of the immune cell infiltration landscape. (**E**) Comparison of estimate score, immune score, StromalScore, and TumorPurity in high- and low-risk groups. (**F**) Correlation of estimate score, immune score, StromalScore, and TumorPurity with patients’ OS in the TCGA-UCEC cohort.

We introduced the model into the full TCGA database for endometrial cancer with clinical information to explore the hidden differences between groups with high and low RNA modification–related risk scores derived from the constructed model, and obtained a high-risk group (*n* = 266) and a low-risk group (*n* = 267) based on RNA modification–related risk score. We performed a survival analysis and produced a Kaplan–Meier graph showing that the high-risk group had significantly worse outcomes (*P* < 0.001) (Fig. 4A). We also introduced the "CIBERSORT," "ssGSEA," and "xCELL" algorithms to further understand the differences in immune microenvironment between the two groups. As shown in the figure, the abundance of most infiltrated immune cells in the high-risk group was lower (P < 0.05), including plasma cells, CD8 T cells, Tregs, dendritic cells, activated B cells, NK cells, central memory CD4 T cells, eosinophils, immature B cells, macrophages, mast cells, MDSCs, monocytes, neutrophils, follicular helper T cells, and others. Of course, some immune cells were more abundant in the high-risk group, including naive B cells, M1 macrophages, and effector memory CD4 T cells (Fig. 4B–D). Further, the overall level of immune cell infiltration was significantly lower in the high-risk group, which might result from the high expression of RNA modification–related genes. The decreased immune cell infiltration also represented a poor prognosis. Next, we introduced the ESTIMATE algorithm to evaluate the immune microenvironment and matrix environment between the two groups. As shown in the figure, the high-risk group had higher stromal, immune, and estimate scores, and lower tumor purity scores (Fig. 4E). We independently conducted survival analysis for the four scores of the ESTIMATE algorithm to explore the connection between them and survival prognosis. It was concluded that the immune and estimate scores were significantly correlated with the prognosis of patients, and the higher the score, the better the prognosis (P < 0.05). However, tumor purity showed an opposite trend, and the stromal score had no significant correlation with the prognosis of patients (Fig. 4F).

### RNA modification-associated risk model and immunotherapy

**Figure 5:**
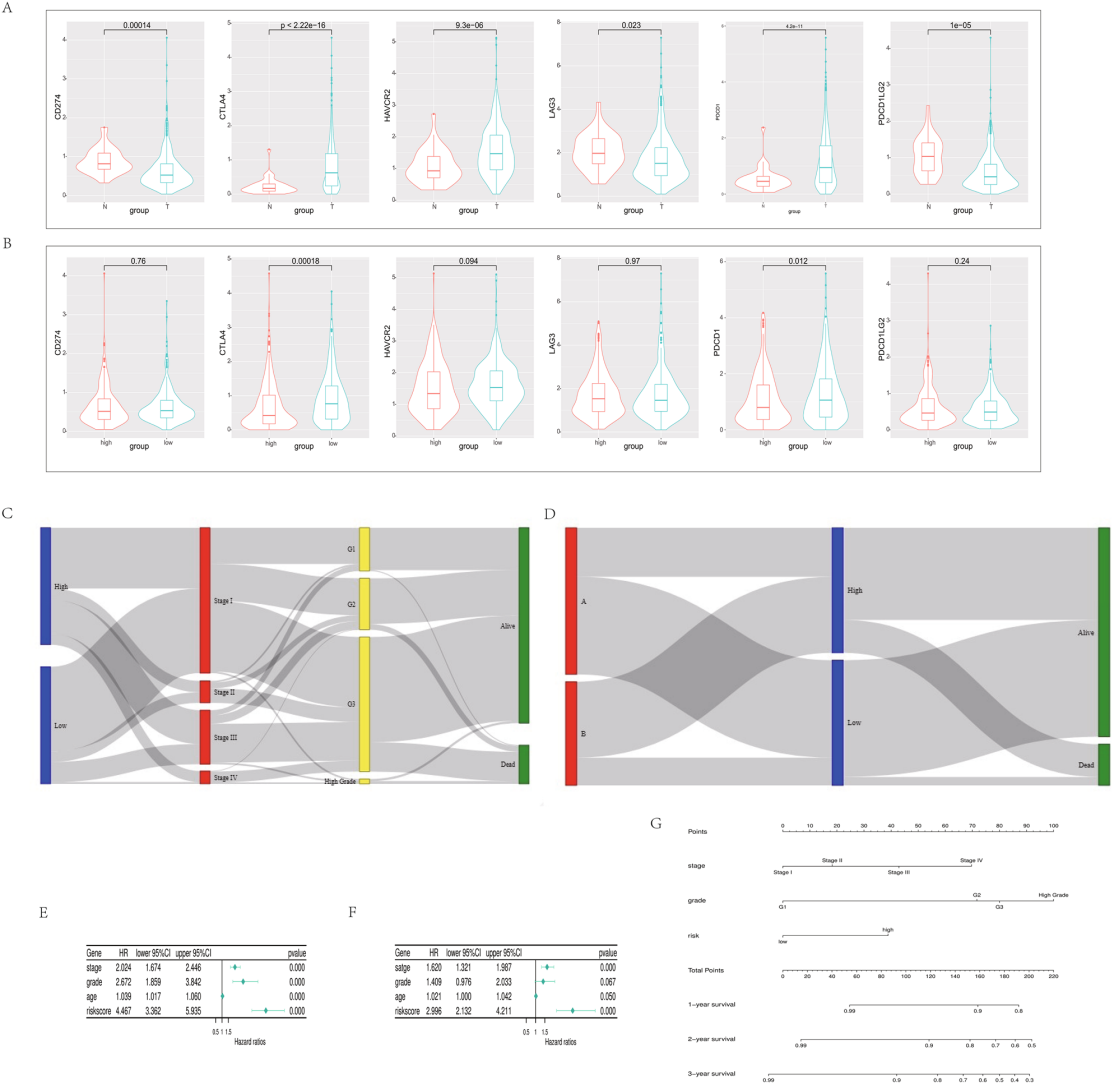
(**A**) Comparison of immune checkpoint expression levels between tumor and normal patients in the TCGA-UCEC cohort. (**B**) Comparison of immune checkpoint expression levels between high and low-risk scores in the TCGA cohort. (**C**) Mulberry map summarizing the high- and low-risk scores in the TCGA cohort. (**D**) Mulberry map summarizing the relationship between RNA-modified cluster, high- and low-risk scores in the TCGA, and survival state. (**E**) Cox univariate regression analysis of stage, grade, age, and risk. (**E**) Correlation between score and OS. (**F**) Cox multivariate regression analysis of the correlation between stage, grade, age, and risk score and OS. (**G**) Comprehensive nomogram for predicting the probabilities of patients with UCEC with 1-, 2- and 3-year OS in the TCGA database.

First, we included the expression profile data of all normal endometrial samples and endometrial cancer samples of TCGA and observed the expression levels of common immune checkpoints, including PDCD1, CD274 (PDL1), PDCD1LG2 (PDL2), CTLA4, LAG3, and HAVCR2. All immune checkpoints were significantly different between the normal and tumor groups (*P* < 0.05), in which PDCD1, CTLA4, and HAVCR2 were highly expressed in tumor tissues, while CD274, LAG3, and PDCD1LG2 were highly expressed in normal tissues (Fig. 5A). Similarly, we introduced the RNA modification–related risk assessment model to classify all tumor samples into a high-risk group and a low-risk group, and compared the expression levels of immune checkpoints between the two groups. PDCD1 and CTLA4 were more highly expressed in the low-risk group; however, the expression levels of CD274, LAG3, HAVCR2, and PDCD1LG2 were not significantly different (Fig. 5B). This gave us the reason to believe that if the model was applied to clinical practice, the return rate of immunotherapy in the low-risk group was higher.

### RNA modification-associated risk model can be used as an independent prognostic factor for UCEC

We combined the group information and clinical features and drew the mulberry chart to more intuitively examine the correlation between the groups of RNA modification–related risk assessment models, the cluster groups, and the clinical features of patients. As shown in the figure, the high-risk group had a higher degree of coincidence with the higher stage and grade and poor prognostic outcomes. The opposite was true for the low-risk group (Fig. 5C). This suggests that there may be a certain correlation between high RNA modification-related risk assessment model scores and higher stage, grade. The high-risk group also had a higher overlap with the RNA modification subtype B group, namely the RNA modification signature high-expression group, while the low-risk group had a higher degree of overlap with the group with low RNA modification signature expression (Fig. 5D). Due to the potential correlations among RNA modification-related risk assessment model scores, stage, grade, and age, all of them may serve as risk factors for poor prognosis in UCEC patients. Many correlations were found among RNA modification–related risk assessment model scores, stages, grades, and ages, and they might all be the risk factors for the poor prognosis of UCEC. Therefore, we attempted to determine using univariate and multivariate Cox regression analyses whether RNA modification–related risk assessment model scores were clinically independent prognostic factors for patients with UCEC. RNA modification–related risk assessment model scores, stage, grade, and age were analyzed as covariables. We observed that age, stage, grade, and RNA modification-related risk assessment model scores were all independent factors that could be used to predict the prognosis of UCEC (Fig. 5E and F). By combining independent prognostic factors, we constructed a column graph to enable clinicians to predict mortality in patients with UCEC. Combining RNA modification-related risk assessment model scores with stage and grade can better evaluate patient prognosis. A total score was obtained by adding the corresponding points of each prognostic factor; the higher the total score, the worse the survival rate was (Fig. 5G).

### Identification of key genes

**Figure 6:**
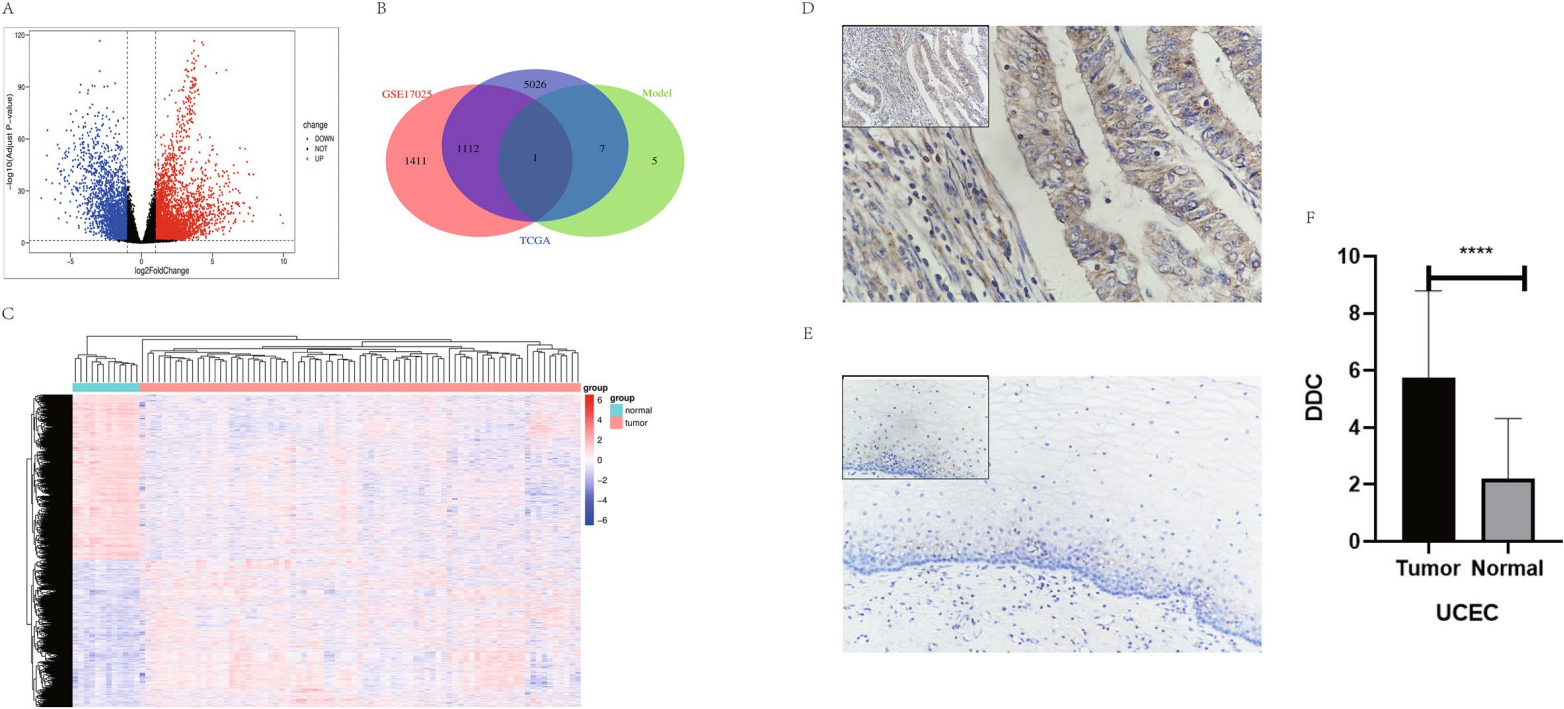
(**A**)Volcanic map of UCEC cancer and paracancer differentially expressed genes (DEGs) of TCGA. (**B**) Heatmap of UCEC cancer and paracancer DEGs of GSE17025 dataset. (**C**) Venn map of LASSO model key genes and DEG construction of the TCGA database and GSE dataset. (**D**) Immunohistochemical results of DDC in UCEC tumor tissue. (**E**) DDC immunohistochemical results of UCEC adjacent tissues. (**F**) Statistical results of immunohistochemical scores of UCEC cancer and adjacent tissues of DDC for 60 pairs.

The DEGs were analyzed according to the expression profiles in the TCGA tumor samples and normal samples, the GEO database GSE17025 was used to screen out DEGs using the "limma" package, and the intersection of the two groups of DEGs and LASSO model genes provided the key gene DDC (Fig. 6A–C).

### Verification of the differential expression of DDC

The IHC results of 60 paired UCEC tissues and adjacent tissues demonstrated high expression of DDC in tumor tissues (5.733 ± 3.052 vs 2.200 ± 2.122, *P* < 0.0001) (Fig. 6D–F).

### Analysis of the potential biological functions of DDC and the relationship between DDC and immune infiltration

**Figure 7:**
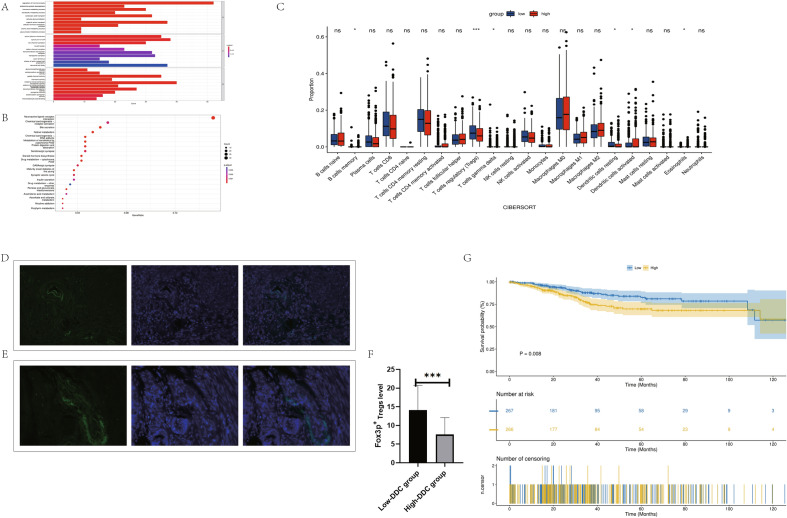
(**A**) GO enrichment analysis of DDC. (**B**) KEGG enrichment analysis of DDC. (**C**) CIBERSORT for evaluating the relationship between DDC and UCEC immune infiltration. (**D**) Immunofluorescence staining of Fox3P-labeled Tregs in the high–DDC expression group. (**E**) Immunofluorescence staining of Fox3P-labeled Tregs in the low-DDC expression group. (**F**) Statistical results of infiltration level of Fox3P-labeled Tregs between high- and low-DDC expression groups. (**G**) According to DDC expression level, the samples were divided into high- and low-DDC expression groups for survival analysis.

The GO enrichment analysis showed that the cellular functions of DDC included the regulation of hormone levels, endocrine system development, hormone metabolism, metabolism of heterogeneous organisms, carboxylic acid transport, organic anion transport, cytohormone metabolism, glucuronic acid metabolism, glucuronic ester metabolism, apical plasma membrane, ion channel complex, and so on. The cationic channel complexes and other related functions were enriched (Fig. 7A). KEGG enrichment showed that the cellular functions of DDC included metabolism of xenobiotics by cytochrome P450, protein digestion and absorption, steroid hormone biosynthesis, drug metabolism, GABAergic synapse, synaptic vesicles, insulin secretion, drug metabolism, and pentose and glucuronate interconversions. Ascorbate was related to aldarate and porphyrin metabolism (Fig. 7B). The CIBERSORT analysis showed that Treg and quiescent dendritic cells were dominant in the low–DDC expression group, while memory B cells, activated dendritic cells, and eosinophils were dominant in the group with high DDC expression (Fig. 7C).

### Correlation between DDC expression level and Tregs density in UCEC

We performed Treg-labeled Foxp3 immunofluorescence staining on tumor tissues of 60 patients with UCEC to confirm the effect of DDC on immune cell infiltration. The results showed that the infiltration level of Foxp3 + Tregs in UCEC tissues (14.08 ± 6.643) was higher in the low–SBSN expression group than in the high-SBSN expression group (7.565 ± 4.551; *P* = 0.001) (Fig. 7D–F).

### Differentiation between DDC and UCEC prognosis

The patients were divided into high- and low-DDC expression groups according to the DDC expression level for survival analysis to explore the correlation between the survival of patients with DDC and those with UCEC. As shown in the figure, the prognosis in the high-DDC expression group was significantly worse than that in the low-DDC expression group (Fig. 7G).

## Discussion

RNA modifications play a significant role in the occurrence and progression of tumors. However, the expression and function of RNA modifications in Uterine Corpus Endometrial Carcinoma (UCEC) have not been thoroughly described. In this study, we conducted clustering based on RNA modification regulatory factors and identified cluster A with low levels of RNA modification regulatory factors, and cluster B with high levels. Patients in cluster A exhibited betted prognosis compared to those in cluster B. Moreover, there were notable differences in the tumor microenvironment between the two groups. We identified differential expression genes (DEGs) based on these DEGs in cluster A and B. Utilizing these DEGs, we developed an RNA modification-related risk score for UCEC patients, which was found to be an adverse prognostic factor with good predictive ability. Consequently, UCEC patients were categorized into high-risk and low-risk groups based on the RNA modification-related risk score. The study found that the high-risk group had lower immune infiltration and a worse prognosis, whereas the low-risk group showed a better response to immunotherapy in the predictions. The risk score was identified as an independent factor that influences the prognosis of UCEC patients. In conclusion, our comprehensive analysis of multiple RNA modifications in UCEC suggests that RNA modification regulatory factors are closely associated with the prognosis and tumor microenvironment of UCEC patients.

RNA modifications, as important regulatory factors in cellular biology, participate in multiple biological processes of immune cells, including development, differentiation, activation, migration, and polarization. RNA modifications regulate immune responses, influence the tumor microenvironment, and promote tumor cell proliferation, metastasis, and immune evasion, playing a key role in tumor development^7,25^. M6A methylation promotes the immunosuppressive characteristics of the tumor microenvironment (TME) and supports tumor proliferation through pathways involving hypoxia, metabolic dysregulation, exosomes, and immune cells^26^. In addition, RNA modification patterns can also influence the immune cell characteristics in the tumor microenvironment, for example, in hepatobiliary malignancies. Higher RNA modification scores are associated with increased infiltration of immunosuppressive cells, as well as poorer prognosis^27^.

Estrogen exerts its influence on gene expression through estrogen receptor alpha (ERα), which includes the regulation of RNA modification regulators. One example is the ability of ERα to control the levels of U34 modification enzymes, thereby determining the levels of U34 modified tRNA, which is essential for mRNA translation^28^. Notably, the estrogen pathway was enriched in group B, which had higher regulators of RNA modification in this study. However, additional research is required to explore the impact of estrogen, mediated by RNA modification regulators, on the prognosis of endometrial cancer. Cluster A group, with lower RNA modification, had better prognosis and more immune cell infiltration. The high RNA modified-related gene risk score group had worse prognosis and poor immune infiltration. The RNA modification-related genes risk assessment model showed that the immune checkpoints PDCD1 and CTLA4 were more expressed in the low-risk group, and the model indicated that the immunotherapy effect of the low-risk group was better. These results suggest that RNA modification have a profound impact on prognosis and immune infiltration in endometrial cancer.

DDC is a catecholamine synthase and androgen receptor (AR) coregulatory protein; it enhances AR activity and differentially regulates the AR regulatory gene^29^ DDC can enhance the expression of the known androgen-induced gene TMEPAI^30^. In premenopausal women, AR is involved in multiple processes, such as uterine endometrial preparation for potential pregnancy and post-menstrual endometrial repair^31–33^. The expression of AR in postmenopausal endometrium is significantly higher compared to the proliferative endometrium, and the expression of AR is significantly higher in metastatic endometrial cancer compared with primary tumours^34^. AR gene variants may increase the risk of UCEC^35^. In postmenopausal women, androgens are converted into estrogen by aromatase, which continuously stimulates the growth and proliferation of endometrium^36,37^ and affects the occurrence and development of UCEC. Testosterone and free testosterone were positively associated with endometrial cancer risk in postmenopausal women^38^. Therefore, Androgen is an important factor in the origin and development of UCEC in postmenopausal women. As a coregulatory protein of AR, DDC may play an important role in the metabolism of the aforementioned hormones, thereby affecting the origin and development of UCEC.

DDC (dopamine decarboxylase) is involved in the metabolism of aromatic amino acids, which may affect the function of the hypothalamus. For example, time-restricted feeding can enhance the digestion and absorption of nutrients, thereby increasing the availability of aromatic amino acids. This increased availability of aromatic amino acids may influence the function of the hypothalamus through the modulation of neuroendocrine effects, especially in regulating feeding patterns^39^. The hypothalamus affects estrogen levels by producing and releasing various hormones. For instance, the hypothalamus can produce gonadotropin-releasing hormone (GnRH), which stimulates the pituitary gland to release gonadotropins, further stimulating the ovaries to produce estrogen. In another study, inhibitors of DDC had an inhibitory effect on basal pulsatile LH secretion, which may be related to the role of DDC in catecholamine synthesis^40^. The secretion pattern of LH directly affects estrogen levels^41^. Therefore, DDC may influence estrogen secretion through the hypothalamus. Studies have shown that excessive estrogen, whether endogenous or exogenous, is associated with an increased risk of endometrial cancer. Estrogen can stimulate the proliferation of endometrial cells, and without the balancing effect of progestogens, it may lead to the occurrence and development of endometrial cancer^42–44^.

The GO and KEGG enrichment analysis showed that DDC affected the regulation of hormone levels, endocrine system development, hormone metabolism, and steroid hormone biosynthesis. These results supported the hypothesis that DDC affected the prognosis of patients with UCEC by affecting hormone metabolism. Several amino compounds comprised the catechol structure, including dopamine, norepinephrine, and epinephrine and their derivatives. Catecholamine (CT) itself is synthesized by DDC^45^. Human macrophages have a CT biosynthetic pathway. CT is a regulatory factor of immune cell proliferation and differentiation^46^. DDC has different effects in different tumors. High expression of DDC in neuroendocrine cancers such as lung carcinoid and small cell lung cancer is associated with worse prognosis, but the overexpression of DDC mRNA in colorectal adenocarcinoma is associated with better prognosis^29^. Using bioinformatics analysis, we found that the low–DDC expression group in endometrial carcinoma had a better survival prognosis.

Subsequently, we found by immunofluorescence that Tregs were dominant in the low–DDC expression group. Tregs, as natural immunosuppressive cells, usually lead to tumor development and metastasis^47,48^, which was inconsistent with better survival prognosis. Resting dendritic cells (DCs) can transform non-regulatory T cells into regulatory T cells through interactions with CD28 and CTLA-4^49^. The low DDC expression group has a higher proportion of resting dendritic cells. Therefore, the high levels of regulatory T cells in low DDC expression group may due to the elevated levels of resting dendritic cells. The higher level of quiescent DCs in the low expression group of DDC may be related to androgens^50–52^, and further investigation is warranted to elucidate the underlying mechanisms by which DDC affects immune cell infiltration.

The DDC inhibitor carbidopa can delay the growth of prostate tumors by reducing the activity of AR through inhibition of the co-activating effect of DDC^18,20^. DDC inhibitors carbidopa and 3-hydroxybenzylhydrazine can irreversibly bind to DDC^53^. These findings suggest that DDC can serve as a biomarker for guiding molecular diagnosis and promote the development of novel individual treatment strategies for UCEC patients. DDC inhibitors may improve the development and prognosis of UCEC by reducing catecholamine production, lowering estrogen levels, and inhibiting androgen receptor activity. Based on the aforementioned results, we boldly speculated that DDC inhibitors might positively affect the treatment of endometrial cancer, which can be investigated in follow-up studies.

## Conclusions

We conducted a comprehensive analysis on the impact of RNA modifications on the prognosis and immune infiltration in Uterine Corpus Endometrial Carcinoma (UCEC). The clustering of RNA modification regulators and risk scoring based on RNA modification-related genes showed strong predictive ability for the prognosis of UCEC patients and profoundly influenced immune infiltration within the Tumor Microenvironment (TME) of UCEC patients. These findings have potential implications for prognosis prediction and selection of targeted therapies in UCEC patients. DDC was identified as a key risk factor in UCEC, and further attention should be given to the related research on DDC inhibitors in UCEC. Moreover, it is important to note that this study evaluated the prognosis using UCEC patient data solely from TCGA. Therefore, future studies should include more external datasets for validation purposes.

### Supplementary Information

Supplementary Information 1.

Supplementary Information 2.

Supplementary Information 3.

Supplementary Information 4.

## Data Availability

All data generated or analysed during this study are included in this article.
